# Near-complete tongue transection during spinal surgery with transcranial MEP monitoring: A case report

**DOI:** 10.1097/MD.0000000000046284

**Published:** 2026-01-02

**Authors:** Jaesuk Kim, Haneul Jeong, So Young Kwon

**Affiliations:** a Department of Anesthesiology and Pain Medicine, The Catholic University of Korea, St. Vincent’s Hospital, Seoul, Korea.

**Keywords:** bite injuries, evoked potentials, intraoperative neurophysiological monitoring, motor, postoperative complications, tongue injuries

## Abstract

**Rationale:**

IONM, especially transcranial MEPs, is a cornerstone in preventing neural injury during spinal surgery. However, transcranial stimulation may result in forceful jaw closure, causing oral trauma such as tongue lacerations, dental injury, or rarely, airway compromise. This case underscores the importance of recognizing and preventing rare but serious complications related to intraoperative neuromonitoring.

**Patient concerns:**

A 55-year-old female with progressive lower limb weakness and thoracic kyphotic deformity underwent multi-level posterior spinal fusion and vertebral column resection.

**Diagnoses:**

A near-complete laceration of the left lateral tongue was noted during emergence from anesthesia, suggesting bite-induced injury from MEP monitoring.

**Interventions:**

The patient underwent immediate otolaryngology consultation and primary repair under general anesthesia. Postoperative management included conservative treatment with topical policresulen and monitoring.

**Outcomes:**

The lesion healed fully without infection or further intervention. The patient was discharged in stable condition.

**Lessons:**

This report highlights a severe yet preventable complication of MEP monitoring. Soft bite block use, careful oral positioning, and intraoperative vigilance – especially in prolonged prone procedures – are essential preventive strategies. Early recognition and timely intervention are crucial for minimizing morbidity.

## 1. Introduction

IONM, comprising both MEPs and SEPs, is a standard practice in complex spinal surgery to decrease the incidence of neurological compromise.^[1,2]^ These monitoring techniques improve intraoperative safety by delivering continuous assessment of motor and sensory tract integrity during spinal cord manipulation.^[1,2]^

In procedures targeting the cervical or upper thoracic spine, transcranial MEP monitoring is particularly important, as these regions are more susceptible to injuries affecting the corticospinal tracts innervating both upper and lower limbs. Through MEPs, surgical and anesthesia teams are afforded real-time evaluation of gross motor function, thereby supporting risk reduction in complex cases.

However, transcranial MEP stimulation may be accompanied by certain complications.^[3,4]^ An often overlooked yet clinically important risk is tongue injury, which can arise from involuntary jaw-closing muscle contractions during high-voltage stimulation. Such events can result in bite trauma, with severity ranging from superficial mucosal abrasions to deep lacerations and hematomas. The reported incidence of tongue injuries related to MEP stimulation is low, ranging from 0.1% to 0.6% in spinal surgeries using neuromonitoring.^[5,6]^ Although minor tongue injuries related to MEP monitoring have been previously documented, to our knowledge, no prior reports have described a near-complete tongue transection caused by transcranial MEP stimulation, making this case exceptionally rare.

In addition to the lack of bite blocks, other contributing factors such as prolonged prone positioning, tongue edema, and absence of intraoral visualization may increase the risk. Thus, preventive strategies should not be limited to bite block usage alone, but also include careful intraoral positioning, consideration of airway adjuncts, and intermittent reassessment during long-duration procedures.

We report a case involving a 55-year-old female with compressive myelopathy and a kyphotic deformity at the upper thoracic level, who sustained a lateral tongue laceration during intraoperative MEP monitoring. This case draws attention to a preventable neuromonitoring complication and emphasizes the critical need for oral protection strategies in patients undergoing high-risk spinal procedures.

## 2. Case presentation

### 2.1. This study adheres to CARE guidelines for case reporting

This study was approved by the Institutional Review Board of St. Vincent Hospital, College of Medicine, The Catholic University of Korea (Institutional Review Board No. VC25ZISE0150).

A 55-year-old female with a longstanding thoracic kyphotic deformity had been managed non-operatively for several years. Over the past 2 years, she experienced worsening bilateral lower extremity weakness, followed by impairment of bowel and bladder function. MRI identified compressive myelopathy associated with a gibbus deformity extending from T4 to T7, leading to her admission for surgical management.

She underwent posterior spinal fusion from D1 to D10, VCR at D5–D6, partial corpectomy at D4 and D7, and decompressive partial laminectomy at D4 and D7. Intraoperative monitoring of MEPs and SEPs was utilized throughout the operation. No intraoperative technical complications occurred.

General anesthesia was administered using total intravenous anesthesia, consisting of continuous propofol and remifentanil infusions. Neuromuscular blockade was induced with 50 mg of rocuronium at the outset, with a supplementary dose of 20 mg given before skin incision. To maintain the integrity of neuromonitoring, no additional muscle relaxants were used. The total duration of surgery was approximately 11 hours and 45 minutes.

An oral endotracheal tube was used for airway management and was fixed at the right oral commissure. There was no displacement or excessive pressure noted around the tongue during intubation or fixation.

**Figure 1.**
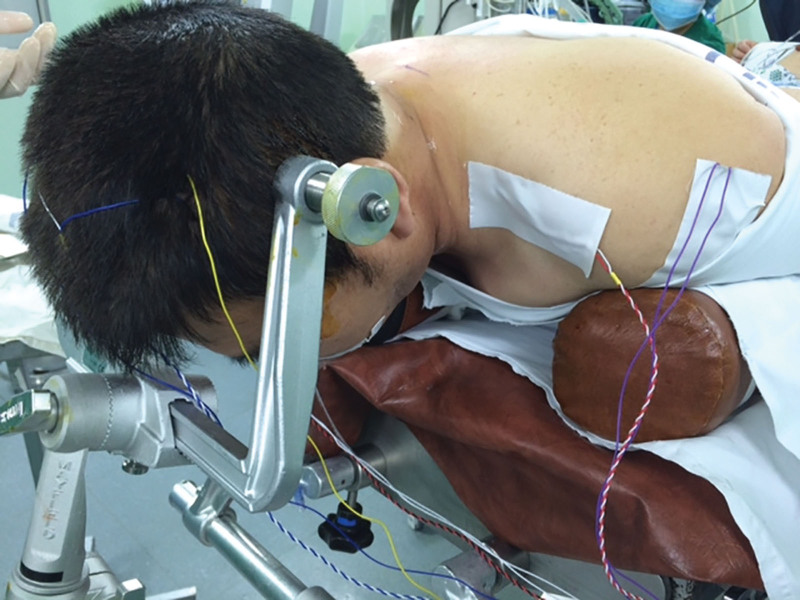
Configuration of transcranial MEP stimulating and recording electrodes utilized during spinal surgery neuromonitoring. (This representative image was sourced from a different patient who followed the identical monitoring protocol, and does not illustrate the patient featured in this case report.) MEP = motor evoked potential.

Transcranial MEPs were elicited using scalp electrodes positioned at the standard C3 and C4 locations in line with the international 10 to 20 EEG system (Fig. 1). In our institution, stimulation is routinely titrated from 300 volts up to a maximum of 400 volts to ensure reliable and reproducible responses. This approach was implemented in the present case. Bite blocks were not utilized during the procedure, consistent with standard practice at our center, where bite protection is not routinely provided except in patients with specific risk factors. Throughout the procedure, MEP signal quality and consistency remained within acceptable limits. Approximately 10 MEP stimulations were delivered during the procedure. Baseline and subsequent responses remained stable, with no significant amplitude reductions or signal loss throughout the surgery.

**Figure 2.**
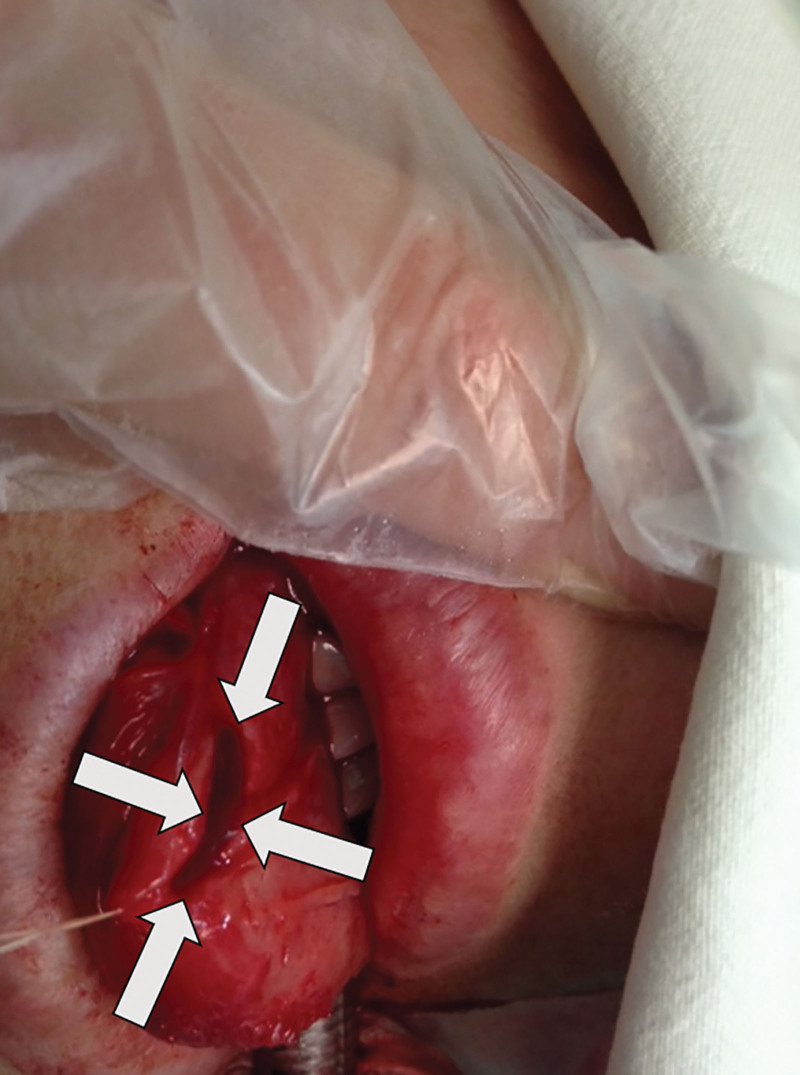
Immediate postoperative presentation: left lateral tongue laceration (highlighted with arrow).

Following the operation, the patient was placed back into the supine position. During emergence from anesthesia, clinicians noted a near-complete laceration (nearly a full-thickness transection) of the left lateral tongue (Fig. 2). An immediate otorhinolaryngology consultation was obtained, and primary suture repair was promptly performed while the patient remained under general anesthesia.

The laceration measured approximately 3.5 cm in length and extended into the intrinsic muscular layer of the tongue without involvement of major vascular structures.

**Figure 3.**
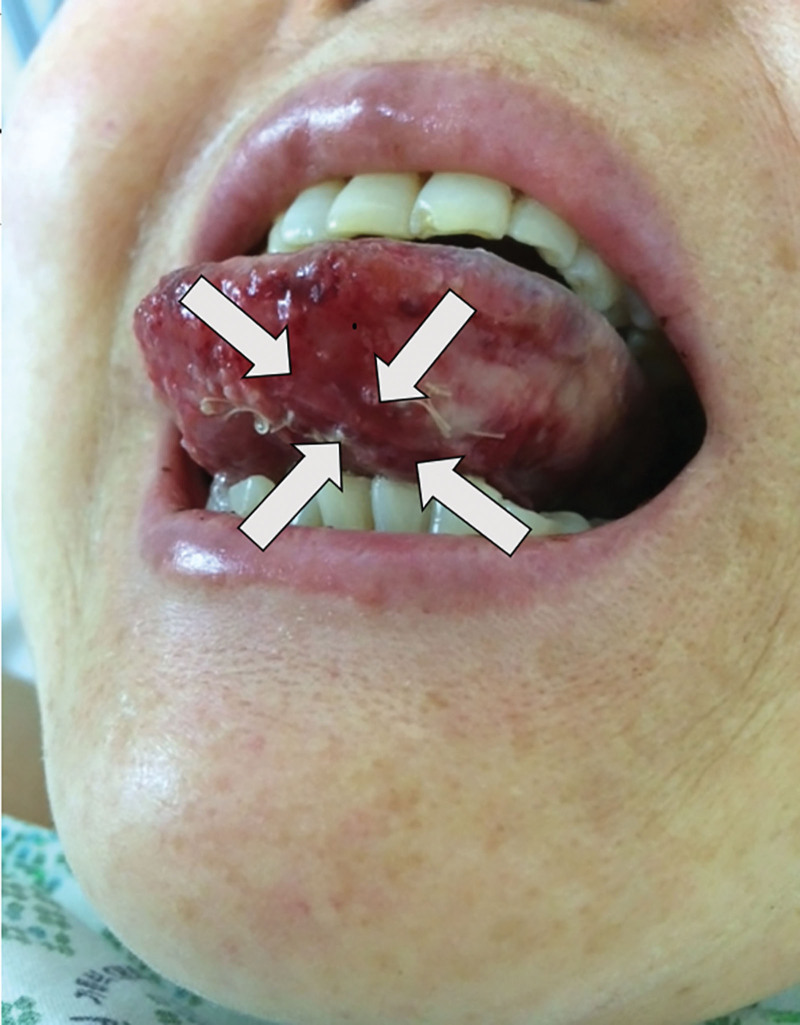
Decreased edema with evidence of mucosal healing (arrow indicates previous injury site).

On postoperative day 1, the patient was reassessed at the otolaryngology outpatient clinic. The evaluation demonstrated mucosal ulceration, submucosal hematoma, and diffuse swelling in the left lateral tongue. Conservative management with topical policresulen (commercially known as Albothyl) along with continued close observation was maintained. Over the following days, the lesion resolved fully, and the patient was discharged in stable condition without evidence of subsequent complications (Fig. 3).

## 3. Discussion

Transcranial MEP monitoring is an important modality for detecting potential compromise of the motor pathways during spinal surgeries, especially those involving deformity correction or VCR.^[2]^ Despite its clinical benefits, the technique is associated with known complications, particularly activation of the masseter and temporalis muscles, which can cause involuntary jaw closure leading to bite-related oral injuries including tongue lacerations, lip trauma, and dental fractures.^[6,7]^

Other complications described include scalp burns from stimulating electrodes, the induction of seizures, and cardiovascular reactions such as transient hypertension or tachycardia, especially in the context of high-voltage stimulation.^[4,8]^ Although uncommon, airway obstruction can result from tongue swelling or hematoma, highlighting the importance of vigilant intraoperative monitoring and timely identification of complications.^[4]^

Although bite-induced injuries have been documented in the literature, to our knowledge, no previous case of a near-complete tongue transection during MEP monitoring has been reported.^[3,6,7]^ The present case thus represents an unusually severe presentation of this rare complication.

In this case, the patient underwent a multi-level thoracic procedure consisting of posterior spinal fusion, corpectomies, and decompressive laminectomies, necessitating aggressive intraoperative neuromonitoring. While the surgical process itself was uneventful, a substantial lateral tongue laceration was observed during emergence from anesthesia. This injury was compatible with bite-induced trauma, likely resulting from transcranial MEP stimulation.

A review of the operative and anesthetic documentation revealed multiple contributing factors to the tongue injury. First, the patient was maintained in the prone position, inherently limiting direct visualization of the face and oral cavity throughout the case. Second, the procedure lasted nearly 10 hours, with prolonged prone positioning potentially predisposing the tongue to edema, thus increasing susceptibility to trauma or compression. Lastly, the absence of a bite block allowed for unprotected mandibular motion during MEP stimulation.

These combined factors resulted in an environment where the edematous tongue, lacking sufficient protection, was inadvertently trapped between the teeth during transcranial stimulation-induced jaw contractions. This case demonstrates the necessity for thorough preoperative planning and diligent intraoperative precautions when utilizing MEP monitoring, particularly for extended procedures in the prone position.

Management of such injuries is predominantly conservative. Local wound care and infection prophylaxis generally suffice.^[9]^ In our experience, the application of topical policresulen and meticulous observation promoted complete spontaneous healing without requiring surgical intervention.

In terms of management, topical policresulen (commercial name: Albothyl) was selected due to its availability in our institution and its established use in promoting mucosal healing and reducing microbial colonization in ulcerated wounds.^[10]^ While not universally adopted, policresulen is widely used in parts of Europe and Asia and has been reported to enhance wound resolution in oral injuries.

Notably, this case illustrates multiple risk factors that must be taken into account during the planning and execution of long-duration spine surgeries under intraoperative neuromonitoring. Extended prone positioning, limited intraoral visualization, and prolonged dependency-induced tongue edema significantly increased the risk of trauma.^[11]^ Additionally, the lack of a soft bite block enabled unprotected mandibular closure during MEP stimulation and ultimately resulted in a preventable tongue injury.

These observations stress the necessity for increased intraoperative vigilance during lengthy surgical procedures, especially those conducted in the prone position. The consistent use of soft bite blocks, particularly in high-risk neuromonitoring settings, should be adopted as standard practice. Furthermore, periodic reevaluation of oral cavity positioning – even in the prone position – may further decrease the risk of bite-related injuries.

Limitations of this report include the inability to conduct direct intraoperative oral cavity inspection due to the prone position, the lack of intraoral photography at the moment of injury, and the fact that this is a single-center experience. Furthermore, institutional variation in IONM protocols may limit generalizability.

To advance patient safety, integrating these strategies into formal surgical and anesthetic guidelines can help reduce preventable complications associated with transcranial MEP monitoring, thereby enhancing outcomes in complex spinal surgeries.

## 4. Conclusion

This case underscores a rare but preventable complication of intraoperative MEP monitoring – severe tongue laceration – resulting from prolonged prone positioning, tongue edema, and the absence of a bite block. While managed conservatively without further sequelae, this event highlights the critical importance of soft bite block application and intraoral protection during lengthy spine surgeries. Heightened intraoperative vigilance and the implementation of basic preventive strategies can significantly enhance procedural safety in neuromonitoring-assisted operations.

## Author contributions

**Conceptualization:** So Young Kwon.

**Investigation:** Jaesuk Kim, So Young Kwon.

**Project administration:** So Young Kwon.

**Resources:** So Young Kwon.

**Writing – original draft:** Jaesuk Kim.

**Writing – review & editing:** Jaesuk Kim, Haneul Jeong, So Young Kwon.
